# Phase III, randomized, open-label study of durvalumab (MEDI4736) monotherapy, or durvalumab + tremelimumab, versus standard of care (SoC), in recurrent or metastatic (R/M) squamous cell carcinoma of the head and neck (SCCHN): eagle

**DOI:** 10.1186/2051-1426-3-S2-P150

**Published:** 2015-11-04

**Authors:** Robert L Ferris, Caroline Even, Robert Haddad, Makoto Tahara, Trishna Goswami, April Franks, Ugochi Emeribe, Anthony Jarkowski, Giovanni Melillo, Lisa Licitra

**Affiliations:** 1 Department of Otolaryngology and Cancer Immunology Program, University of Pittsburgh Cancer Institute, Pittsburg, PA, USA; 2 Institut Gustave-Roussy, Villejuif, France; 3 Dana Farber Cancer Institute, Boston, MA, USA; 4 National Cancer Center Hospital East, Kashiwa, Japan; 5 AstraZeneca, Gaithersburg, MD, USA; 6 Fondazione IRCCS Instituto Nazionale dei Tumori, Milan, Italy

## Background

Patients with R/M SCCHN have a poor prognosis, and current therapies used after failure of first-line platinum-based chemotherapy provide transient, limited benefit. SCCHN tumors are highly immunosuppressive and evade immune detection by exploiting inhibitory immune checkpoints such as the programmed cell death ligand-1 (PD-L1)/programmed cell death-1 (PD-1) axis. High mutational load and their relationship to human papillomavirus (HPV) infection may make these tumors amenable to immunotherapy. Durvalumab is a selective, human IgG1 mAb that blocks binding of PD-L1: to PD-1 (IC_50_ 0.1 nM) and CD80 (IC_50_ 0.04 nM). Tremelimumab is a selective human IgG2 mAb inhibitor of cytotoxic T-lymphocyte-associated antigen-4 (CTLA-4). The PD-1 and CTLA-4 pathways are non-redundant and targeting both induces synergistic antitumor effects, according to preclinical data, and was found to be active and tolerable in a Phase Ib study in patients with NSCLC (NCT02000947). Durvalumab monotherapy has also shown preliminary antitumor activity in a Phase I/II study in patients with solid tumors, including a SCCHN cohort (NCT01693562). In a comprehensive clinical development program of durvalumab in SCCHN, the Phase III EAGLE study (NCT02369874) will investigate the efficacy and safety of durvalumab as monotherapy or in combination with tremelimumab versus SoC.

## Methods

**Figure 1:**
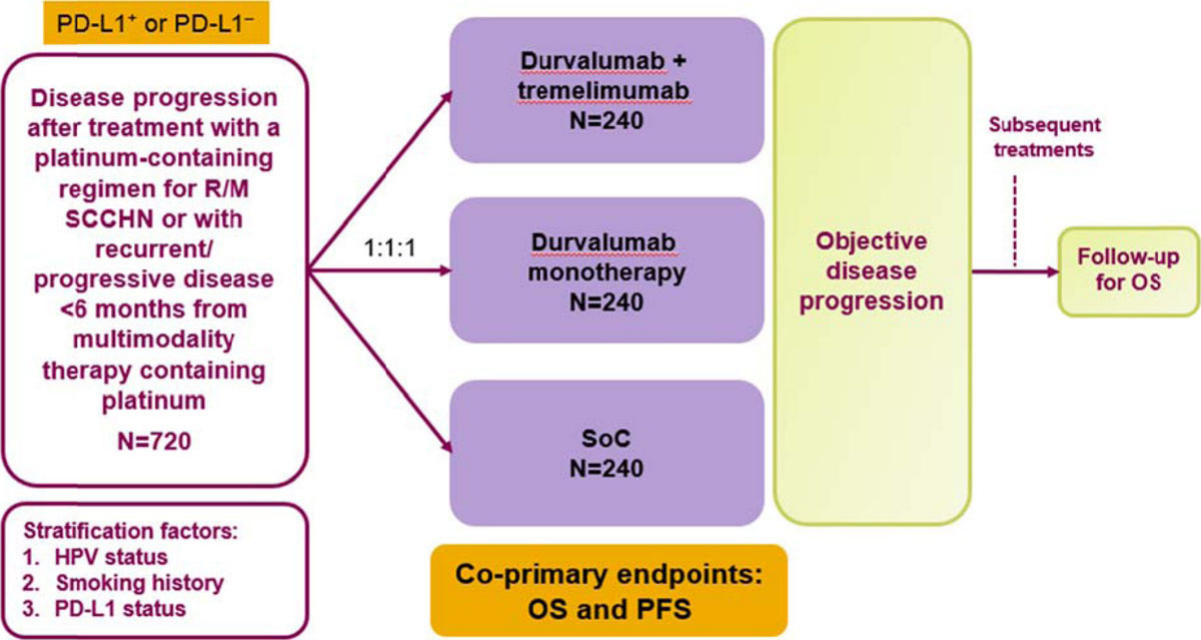


In this Phase III, open-label, multicenter, international study, 720 patients with PD-L1^+^ and PD-L1^–^ R/M SCCHN will be randomized (1:1:1) to receive durvalumab (10 mg/kg IV for up to 12 months); tremelimumab (1 mg/kg IV) plus durvalumab (20 mg/kg IV for up to 12 months); or SoC (cetuximab, taxane, methotrexate, or fluoropyrimidine) (Figure 1). Stratification factors include PD-L1 status, HPV status and smoking history. Eligible patients are immunotherapy naïve and have progressed during or after treatment with a platinum-containing regimen for R/M disease or progressed within 6 months of multimodality therapy containing platinum. Co-primary endpoints are progression-free survival (PFS; RECIST v1.1), based on independent central review and overall survival (OS). Secondary outcomes will assess objective response rate, disease control rate, duration of response, and proportion of patients alive and progression free at 6 and 12 months (using RECIST v1.1 and immune-related RECIST criteria); safety (CTCAE v4.03) and tolerability; and health-related quality of life. Exploratory outcomes include pharmacokinetics, immunogenicity, and potential biomarkers that may influence the progression of cancer and/or prospectively identify patients likely to respond to treatment. Recruitment is underway.

## Trial registration

ClinicalTrials.gov identifier NCT02369874.

